# Backbone and methyl side-chain resonance assignments of the single chain Fab fragment of trastuzumab

**DOI:** 10.1007/s12104-024-10177-3

**Published:** 2024-05-08

**Authors:** Donald Gagné, James M. Aramini, Yves Aubin

**Affiliations:** 1 Centre for Oncology, Radiopharmaceuticals and Research, Biologics and Radiotherapeutic Drugs Directorate, Health Canada, 251 Sir Frederick Banting Driveway, Ottawa, ON K1A 0K9 Canada; 2 Department of Molecular Genetics, University of Toronto, Toronto, ON M5S 1A8 Canada; 3 Department of Chemistry, Carleton University, 1125 Colonel By Drive, Ottawa, ON K1S 5B6 Canada

**Keywords:** Monoclonal antibody, Trastuzumab, NMR spectroscopy, Fragment antigen binding, *Escherichia coli*

## Abstract

Trastuzumab is a therapeutic monoclonal antibody developed to target human epidermal growth factor receptor 2 (HER2) present at higher levels in early cancers. Here we report the near complete resonance assignment of trastuzumab-scFab fragment backbone and the methyl groups of isoleucine, leucine and valine residues, as well as their stereo-assignments. The antibody fragment was produced using a single chain approach in *Escherichia coli*.

## Biological context

Human epidermal growth factor receptor type 2 (HER2) is a member of the superfamily of four receptor types (HER1, HER2, HER3, and HER-) involved in epithelial cell growth and differentiation. Ligand binding to the extracellular domain of the HER receptor induces the formation of homodimers or heterodimers, which in turn activates their intracellular tyrosine kinase domain. Then HER receptor will phosphorylate its dimer partner which will initiate a cascade of events leading to cell proliferation and differentiation(Patel et al., 2020a). Amongst all receptors, HER2 is the only receptor type that has no known ligands, while it is the preferred dimerization partner with other HER types. Overexpression of HER2, is observed in 20–30% of early and advanced breast cancer mainly, but also in advanced stomach cancer and gastro-oesophageal junction cancer(Patel et al., 2020b). In breast cancer cells, the high copy number of HER2 receptors can produce ligand-independent heterodimer formation with HER3 that activates this signaling pathway. HER2-positive cancers, especially breast cancer, have poor clinical prognosis. Trastuzumab (brand name Herceptin® and a number of biosimilars Herzuma®, Kanjinti®, Trazimera®, Ogivri®, Zercepac®, Trastucip®) is a therapeutic monoclonal antibody (mAb) that was developed to target HER2. It binds to the extracellular juxtamembrane domain of HER2 receptor to prevent the activation of its intracellular tyrosine kinase thereby inhibiting the proliferation and survival of HER2-dependent tumors(Hudis 2007). Trastuzumab binding to HER2 inhibits the ligand-independent HER2-HER3 heterodimer formation and HER3 phosphorylation. This suppresses AKT (Protein kinase B) phosphorylation thereby deactivating the phosphatidylinositol 3-kinase (PI3K)/AKT signaling(Yakes et al., 2002) pathway This pathway is highly activated in various types of cancer(Nicholson and Anderson 2002; Carmona et al., 2016). Moreover, antibody-dependent cell-mediated cytotoxicity (ADCC) is one of the main mechanisms of the anti-tumor function of trastuzumab which is mediated by effector immune cells such as natural killer cells (Kim et al., 2017; Tian et al., 2017). Binding of its fragment crystallizable gamma receptors to the antibody Fc fragment initiates the ADCC process leading to the destruction of tumor cells by the immune system. Trastuzumab is a humanized mAb of the immunoglobulin G1 (IgG1) class where the residues involved in antigen binding that form the complementary-determining region (CDR), are from mouse while all constant regions are from human IgG1.

NMR methods at natural abundance have been proposed for the assessment of the higher order structure of mAbs and their Fab and Fc fragments(Brinson et al., 2019; Hodgson et al., 2019). However, the lack of resonance assignment limits the level of NMR characterization of these high molecular weight proteins (49–50 kDa for both Fab and Fc fragments). This has been impeded by the challenge of producing isotopically labelled, in particular highly deuterated, mAb fragments. Recently, that limit has been crossed. Solomon and coworkers reported the backbone resonance assignment of yeast produced NIST-mAb Fab(Chao et al., 2023; Solomon et al., 2023). However, more complex labelling schemes with yeast, such as methyl labelling, are not straightforward and thus require further developments. In parallel, we developed a method using *Escherichia coli* to produce isotopically labelled mAb Fab fragments for NMR resonance assignment (Gagné et al., 2023). The method, is based on the production of a single polypeptide chain in inclusion bodies constructed by the fusion of the heavy and the light chains with a removable linker to facilitate protein refolding. Production of labelled Fab fragments using this method afforded many advantages. All amide deuterons are readily exchanged with protons during the refolding procedure. *E. coli* allows easy isotope incorporation and various labelling schemes such as methyl labelling and surprisingly higher protein yields of 99% deuterated samples. Here we present the backbone and side chain methyl group assignment of isoleucine, leucine and valine residues, including the stereo assignments of leucine and valine methyl groups, of the single chain Fab fragment of trastuzumab.

## Methods and experiments

### Expression and purification of trastuzumab-scfab

The amino acid sequence used for the production of samples of labelled trastuzumab-scFab has been described previously (Gagné et al., 2023). The heavy chain of the Fab domain, residues Glu1 to Pro230 (underlined) where Cys229 has been mutated to Ala229, was linked to residues Asp1 to Cys214 of the light chain via a linker made of five (GGGGS) elements plus SSGLVPRGS. The last residues of the linker contain a thrombin recognition site (LVPRGS). A poly-histidine tag (MGSSHHHHHH HHHHSSGHMLVPR) is fused to the amino terminus of this polypeptide. Thrombin cleavage only removed the fusion tag leaving the linker intact. No attempts were made to cleave the linker with papain post-thrombin cleavage of the tag. We therefore elected to carry out the assignment of trastuzumab-scFab fragment with the following sequence:

- 1-GS*EVQLVESG GGLVQPGGSL RLSCAASGFN IKDTYIHWVR QAPGKGLEWV ARIYPTNGYT*
- 61-*RYADSVKGRF TISADTSKNT AYLQMNSLRA EDTAVYYCSR WGGDGFYAMD YWGQGTLVTV*
- 121-*SSASTKGPSV FPLAPSSKST SGGTAALGCL VKDYFPEPVT VSWNSGALTS GVHTFPAVLQ*
- 180-*SSGLYSLSSV VTVPSSSLGT QTYICNVNHK PSNTKVDKKV EPKSCDKTHT AP***GGGGSGGG**
- 241-**GSGGGGSGGG GSGGGGSGGG GSSSGLVPRG S***DIQMTQSPS SLSASVGDRV TITCRASQDV*
- 301-*NTAVAWYQQK PGKAPKLLIY SASFLYSGVP SRFSGSRSGT DFTLTISSLQ PEDFATYYCQ*
- 361-*QHYTTPPTFG QGTKVEIKRT VAAPSVFIFP PSDEQLKSGT ASVVCLLNNF YPREAKVQWK*
- 421-*VDNALQSGNS QESVTEQDSK DSTYSLSSTL TLSKADYEKH KVYACEVTHQ GLSSPVTKSF*
- 481-*NRGEC*

Expression of labeled ^2^H-^13^ C-^15^N-trastuzumab-scFab was carried out by incubating *Escherichia coli* BL21(DE3) harboring the Histag-trastuzumab-scFab construct (Gagné et al., 2023) at 37℃ (225 rpm) in M9/D_2_O minimal media supplemented with 2 g/L ^2^H^13^ C -glucose and 3 g/L ^15^N-ammonium chloride as the sole source of carbon and nitrogen. Briefly, one colony was transferred to 4 mL of Luria Broth Miller (LB) and incubated for 2 h at 37℃ (225 rpm). A 500 µL aliquot of the LB pre-culture was transferred to 50 mL of M9/H_2_O for another 6 h of incubation after which, 2 mL was transferred into 200 mL of M9/D_2_O for an overnight pre-culture. The following day, the content was transferred to 2 liters of M9/D_2_O and returned to the incubator. Induction with 1 mM thio-D-galactopyranoside (IPTG) was conducted when the OD_600_ was 0.67. After 24 h of expression, cells were recovered by centrifugation at 3,011 x g and stored at -80℃ until used.

Expression of labeled ^2^H-^13^ C-^15^N-^1^H-methyl-(Ile, Leu, Val)-trastuzumab-scFab was conducted as described above, with the exception that 25 mg of α-ketobutyric acid-^13^C-3,3-d_2_ and 50 mg of α-ketoisovaleric-U-^13^C_5_ acid-3-d_1_ (dry powder) were added directly into the culture at an OD_600_ of 0.38, while the induction was conducted with 1 mM IPTG at an OD_600_ of 0.77.

Fractionally labeled ^13^C(10%)-^15^N-trastuzumab-scFab was obtained by expressing the protein in a mixture of 10% ^13^C6 glucose and 90% un-labeled glucose as the sole carbon source.

Protein purifications were conducted using a fast dilution approach at pH 9.0 and with 2 M L-arginine, as described in previously (Gagné et al., 2023). His tag was removed by incubating the protein with 100 units/mg of thrombin in phosphate buffer at pH 7.0 for 5 h at 37℃ under light agitation. Thrombin and Histag were removed with a Hitrap SP (5 mL), followed by size exclusion chromatography using HiLoad 26/60 Superdex 75 pg. Protein yield (before cleavage) of 37, 44, and 36 mg/L of culture were obtained for ^2^H-^13^ C-^15^N-trastuzumab-scFab, ^1^H-I(δ1)LVmethyl-^2^H-^13^ C-^15^N-trastuzumab-scFab, and ^1^H^13^ C(10%)-^15^N-trastuzumab-scFab, respectively. Sample for resonance assignment contained 395 µM ^2^H-^13^C-^15^N-trastuzumab-scFab (21 mg/mL) in 20 mM sodium acetate-d_3_ at pH 5.0 with 5% v/v deuterium oxide for lock frequency purposes in 50 µL and transferred in a 1.7 mm tube. Sample for side-chain methyl groups, prepared as described above with the addition of labelled intermediates as described by Goto and coworkers (Goto et al., 1999), contained 400 µM ^1^H-I(δ1)LVmethyl-^2^H-^13^C-^15^N-trastuzumab-scFab in 300 µL (5 mm Shigemi tube) in the same buffer.

### NMR experiments

Data were collected at 40℃ (313 K) on Bruker AVANCE NEO 600 MHz (side-chain assignment), AVANCE III-HD 700 MHz (backbone assignment) and AVANCE NEO 1 GHz NMR spectrometers equipped with 5 mm, 1.7 mm, and 5 mm, respectively, TCI cryogenically cooled triple resonance inverse probeheads fitted with z-axis gradients. Chemical shift resonances were referenced with sodium 2,2-dimethyl-2-silapentane-5-sulfonate (DSS).

Data collection for the assignment of the backbone resonances used the TROSY-based version(Eletsky et al., 2001) of the standard pulse sequences with deuterium decoupling during carbon evolution from the Bruker library: 2D-^15^NHSQC (trosyetf3gpsi), 3D-HNCO (trhncogp2h3d) (Salzmann et al., 1998), 3D-HN(CA)CO(Clubb and Wagner 1992), 3D-HNCA (trhncagp2h3d2), 3D-HN(CO)CA (trhncocagp2h3d) (Eletsky et al., 2001), 3D-HNCACB (trhncacbgp2h3d), 3D-HN(CO)CACB (trhncocacbgp2h3d) (Grzesiek and Bax 1993; Eletsky et al., 2001) on the 700 MHz NMR spectrometer fitted with the 1.7 mm NMR probehead. All proton-nitrogen planes were collected using a spectral width (SW) of 18 ppm with 2048 real points (^1^H) and 40 ppm with 64 real points (^15^N). The ^13^C indirect dimensions were collected with a SW of 14 ppm, and 128 real points for HNCO/HNCACO, a SW of 30 ppm with 128 real points for HNCA/NHCOCA, and a SW of 80 ppm with 128 real points for HNCACB/HNCOCACB. The acquisition time of 13 h (46 h) for the HNCO (TROSY) and 52 h for all other 3D experiments was used.

Side-chain methyl resonance of isoleucines (delta), leucines and valines were assigned with pulse sequences based on a carbon TOCSY element to transfer the methyl carbon magnetization down to either the alpha carbon or carbonyl prior to transfer to the bonded nitrogen and then proton for detection. These experiments are most efficient at fields of 600 MHz or less. Data were collected on a sample of ^1^H-I(δ1)LVmethyl-^2^H-^13^ C-^15^N-trastuzumab-scFab at 465 µM (25 mg/mL) in 20 mM sodium acetate-d3 at pH 5.0 with 5% v/v deuterium oxide, in a 5 mm Shigemi tube. NMR pulse sequence codes were graciously provided by Prof. Lewis Kay (University of Toronto). In total a series of four 3D experiments 3D-CCC(CO)NH, 3D-HCC(CO)NH, 3D-CCC(CA)NH, and 3D-HCC(CA)NH were acquired (Tugarinov and Kay 2003). Data were collected with a SW of 16 ppm with 2048 real points in the proton direct dimension and a SW of 40 ppm with 64 real points in the nitrogen dimension centered at 120 ppm. The indirect proton dimensions (HCC-) were collected with a SW of 3 ppm with 64 real points and the indirect carbon dimensions (CCC-) with a SW of 22 ppm with 54 real points for a total acquisition time of 54 h and 64 h, respectively. Stereoassignment of the Pro-R and Pro-S of methyl groups of valine and leucine side chains was carried out following the method of Neri et al. (Neri et al., 1989). Briefly, a constant time 2D-^1^-^13^ C CT-HSQC was recorded on a ^1^H^13^ C(10%)-^15^N-trastuzumab-scFab sample using a constant time of 28 ms during carbon evolution. The resulting spectrum provided resonances of opposite phases for the Pro-R and Pro-S methyl groups (Tugarinov and Kay 2004).

### Data analysis and resonance assignment and validation

NMR data were processed using nmrPipe (Delaglio et al., 1995) that was run via the NMRBox web facility (Maciejewski et al., 2017) and visualized with NMRViewJ (Johnson and Blevins 1994). Semi-automated sequential assignment was carried out with the Runabout tool of NMRViewJ software.

### Validation of NMR assignment

The web server of I-PINE (http://i-pine.nmrfam.wisc.edu/index.html) (Lee et al., 2019) was used to verify and validate the trastuzumab-scFab assignments. All peak lists from TROSY-based NMR experiments, namely 2D-^15^N-HSQC, 3D-HNCO, HN(CA)CO, HNCA, HN(CO)CA, HNCACB, and HN(CO)CACB, were used as input files supplemented with the Runabout-manual assignment list and the three-dimensional X-ray structure (PDB ID 5xhg). The output of the server allowed the identification and correction of a few assignments, and the identification of new assignments. In addition, we tested the new assignment protocol BARASA (Bishop et al., 2023) running under NMRBox using the same peak list and assignments as input data used for I-PINE. A total of 20 runs were conducted, using 80 concurrent threads with 0.99 convergence p-value, and a stepwise energy drop of -100. Ca, Cb, and CO zero points were all set to 0.20, with a chemical shift energy range of -50 to 100.

Secondary structure predictions based on using ^13^Cα, ^13^Cβ, ^13^CO, and ^15^N chemical shift resonances of ^2^H-^13^ C-^15^N-trastuzumab-scFab were performed using the web server of CIS 3.0 (Hafsa et al., 2015) (http://csi3.wishartlab.com/cgi-bin/index.php) and TALOS-N (Bartels et al., 1996) inside NMRBox (Maciejewski et al., 2017). Secondary structure elements predicted from chemical shifts were compared to the X-ray structure (PDB ID 5xhg). The validated assignments were deposited in the BioMagResBank under accession number 52,228.

### Extent of assignments and data deposition

#### Resonance assignment of backbone atoms

**Fig. 1:**
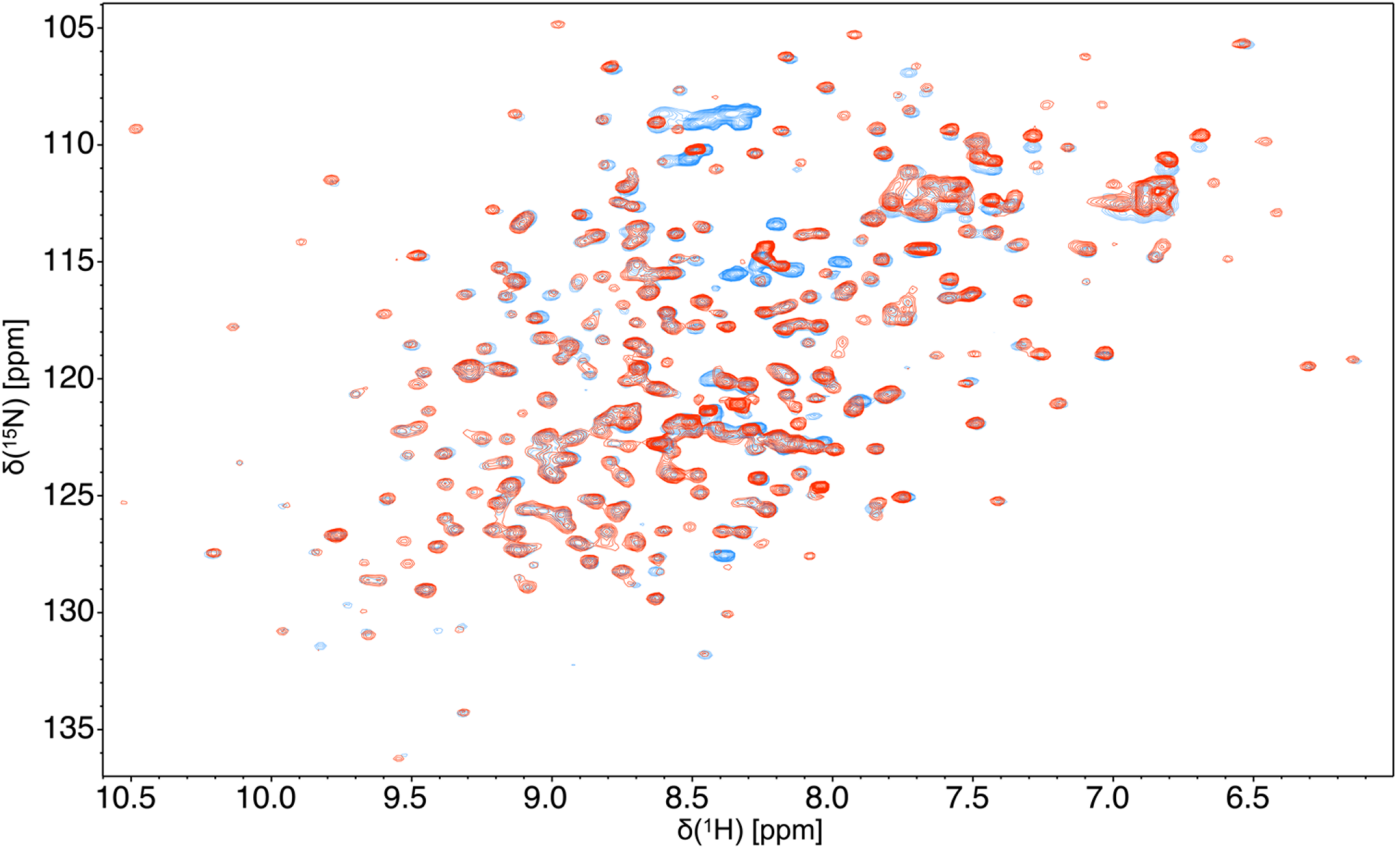
Comparison of trastuzumab-scFab (red, thrombin cleaved, linker present) and trastuzumab-Fab (blue, papain cleaved). The 2D-^1^H-^15^N-SOFAST-HMQC spectrum overlay recorded at 700 MHz at 50ºC indicates that all backbone resonances of the scFab (linker present)

Attempts to remove the linker after thrombin cleavage of the fused poly-histidine tag yielded significant loss of sample. Therefore, we compared the 2D-^1^H^15^ N HSQC of trastuzumab-scFab with the fully cleaved trastuzumab-Fab. The extra resonances belonging to the linker (scFab) were well resolved from any backbone resonances of the Fab and all backbone resonances of both samples were overlapped, indicating that the assignment of the scFab can be directly used for the Fab (Fig. 1). The fragment contained a total of 485 amino acids (50.8 kDa) with the heavy chain having 230 residues (13 prolines) and the light chain having 214 residues (12 prolines).

**Fig. 2:**
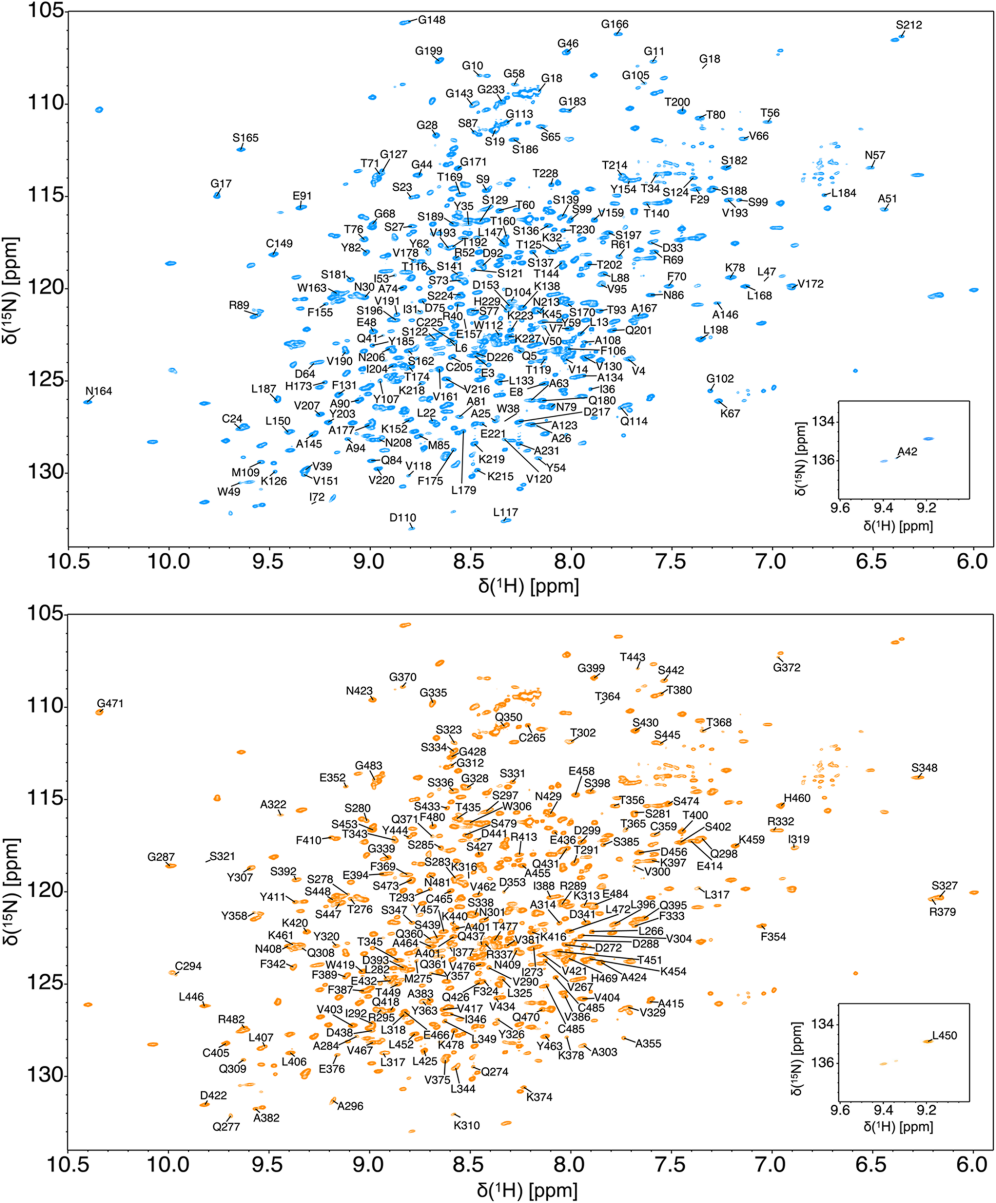
Two-dimensional ^1^H-^15^N-TROSY-HSQC spectrum of ^1^H-I(δ1)LVmethyl-^2^H-^13^C-^15^N-trastuzumab-scFab acquired at 1 GHz at 40℃. The backbone amide chemical shift assignment of heavy and light chains is shown in blue and orange, respectively. The low field region of the spectrum is shown in the bottom-right box

The ^2^H-^13^C-^15^N two-dimensional TROSY HSQC spectrum of trastuzumab-scFab shows well-dispersed resonances, typical of well-folded Fab (Fig. 2). A total of 405 (96.7%) ^1^H^15^ N backbone peaks were assigned, with 204 (94.0%) and 201 (99.5%) in the heavy and light chains, respectively. Assigned carbons include 416 (93.7%) ^13^CO, 422 (95.0%) ^13^Cα, and 373 (91.0%) non-glycine ^13^Cβ. From the 41 residues in the linker, the first two glycines were assigned and the last seven residues including the thrombin site. The Fab fragment is composed of four immunoglobulin domains that are each stabilized by one disulfide bond: Cys24-Cys98 (heavy chain, V_H_), Cys149-Cys205 (heavy chain, C_H_1), Cys294-Cys359 (light chain, V_L_), Cys405-Cys465 (light chain, C_L_), and one bond that links the heavy to the light chain Cys225-Cys485. All cysteine Cb chemical shifts are higher than 35 ppm, which is indicative of properly formed disulfide bonds, while reduced cysteine would have chemical shifts less than 35 ppm (Schulte et al., 2020).

### Resonance assignment of isoleucine delta-1, leucine and valine methyl groups

**Fig. 3:**
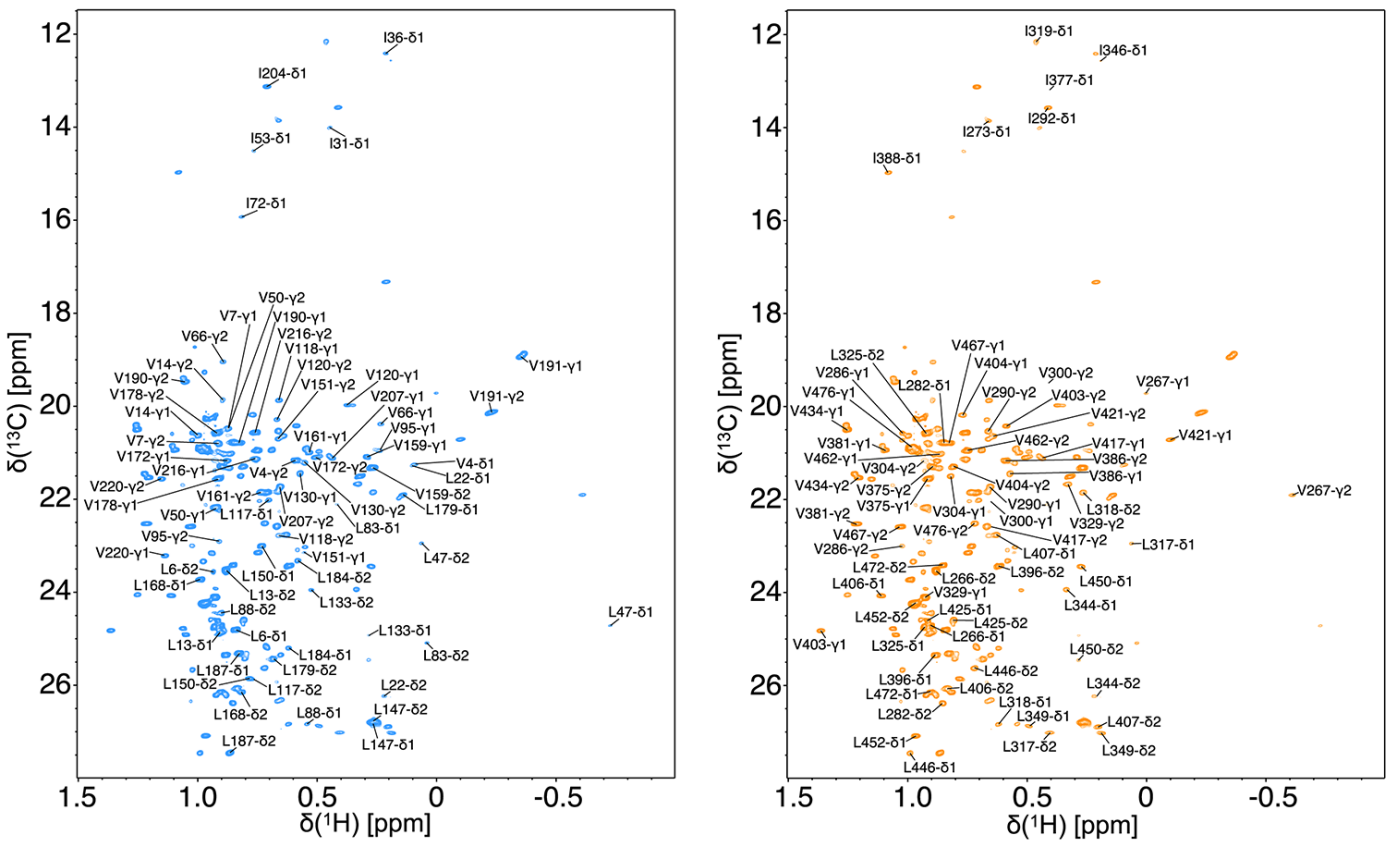
Two-dimensional ^1^H-^13^C CT-HSQC spectrum recorded at 1 GHz of the methyl spectral region of trastuzumab-scFab. The spectrum was acquired using a ^1^H-I(δ1)LVmethyl-^2^H-^13^C-^15^N-trastuzumab-scFab sample, with acquisition at 40℃ , with 28 ms constant-time delay. The methyl chemical shift assignment of isoleucine-δ1, leucine, and valine of heavy and light chains is shown in blue and orange, respectively. Stereospecific NMR assignment of the valines and leucines was conducted with 10% ^13^C and 90% unlabeled carbon as the sole carbon source. Pro-R (γ1 of valine and δ1 of leucine) and Pro-S (γ2 of valine and δ2 of leucine) were determined using 14 and 21 ms of constant-time delays

Using the carbon TOCSY versions of experiments, a total of 11 isoleucines (100%), 28 leucines (93%), and 35 valines (92%) were assigned (Fig. 3). Only four residues have not been assigned, namely Leu20, Leu198, Val93, Val193.

Analysis of the 2D ^1^H^13^ C constant time HMQC experiment on a 10% ^13^C-labeled (90% natural abundance) sample, provided complete stereoassignment for all 28 leucine and 35 valine methyl groups. We used the 1983 IUPAC-IUC recommendation for the identification of the stereospecificity of methyl groups, with Pro-R and Pro-S being identified as γ1 or δ1, and γ2 or δ2 for valine and leucine, respectively (Markley et al., 1998).

### Comparison with NISTmAb assignment

Trastuzumab and the NIST-mAb are two monoclonal antibodies of the IgG1 class with light chain kappa. They both share identical primary sequences in their constant heavy 1 (C_H_1) and constant light domain (C_L_). In order to further validate our assignment, we compared it to the resonance assignment of backbone atoms of the yNISTmAb-Fab(Solomon et al., 2023). It is expected that resonances arising from amides with the same local magnetic environment will have the same chemical shifts while others that have similar or slightly different environments will produce slightly or significantly different chemical shifts. Indeed, comparison of assigned amide groups with same or very similar chemical shifts from both mAbs yielded the same assignment.

### Validation of the backbone assignment

I-PINE was used to validate the current assignment and to help in identifying non-assigned residues. From a total of 419 non-proline residues, I-PINE assigned 403 (96.21%) residues thus providing 15 new assignments. From this total, 370 (95.4%) matched our assignment. The Linear Analysis of Chemical Shifts (LACS) identified 2 outliers: Ser19 in ^13^C-O, and Val95 in ^13^Cα and ^13^Cβ. All cysteines are fully oxidized, with the exception of Cys98, with 74.9% oxidation. Prolines’ isomerization state is mostly trans, with the exception of Pro158, Pro279, and Pro412 consistent with the X-ray structure. Predominantly trans prolines are associated with folded proteins (Alderson et al., 2018).

Initial attempts using BARASA using default parameters to validate the assignment led to poor results: only 195 (46.5%) residues out of the 419 were identified (Table 1). However, optimization of the parameters increased the number of assigned residues to 361 (86.2%), corresponding to 77.4% and 95.5% of the residues of the heavy and light chains, respectively. While BARASA did not provide a higher number of assigned residues, the approach did allow the identification of errors or glitches in the semi-automatic assignment performed with RunAbout such as misinterpretation of which resonance belonged to the Cβ(i) vs. Cβ( i-1) etc.

**Table 1:** Parameters optimization in BARASA

Stepwise energy drop	-100	-500	-1000	-2000
Convergence p-value: 0.99; Min. chemical shift energy: -50				
No. of assignments^1^	361	351	333	328
Matching assignments^2^	335 (92.8%)	326 (92.9%)	312 (93.7%)	309 (94.2%)
Convergence p-value	0.50	0.99		
Stepwise energy drop: -2000; Min. chemical shift energy: -50				
No. of assignments^1^	195	328		
Matching assignments^2^	187 (95.9%)	309 (94.2%)		
Min chem. shift energy	-50	-100		
Stepwise energy drop: -2000; Convergence p-value: 0.99				
No. of assignments^1^	328	344		
Matching assignments^2^	309 (94.2%)	318 (92.4%)		

*Note* Default parameters: stepwise energy drop: -2000; convergence p-value: 0.99; Min. chemical shift energy: -50

1. The number of assignments corresponds to the results returned from the calculations

2. Matching assignment between BMRB 52,228 and the results returned from BARASA

In order to test the ability of BARASA to perform *de novo* resonance assignment of trastuzumab-Fab, we used a SHIFTX2 predicted list of backbone chemical shifts and a mix of ‘known’ and predicted chemical shifts. The list of known chemical shifts were built from residues of the constant domains C_H_1 and C_L_ of trastuzumab-Fab and NIST-Fab that share the same chemical shifts. All remaining (unknown) residues were predicted with SHIFTX2. Finally, a test with SHIFTX2 predicted chemical shifts was carried out. The results (Table 2) showed that both approaches produced good assignments (over 88%) with a very good assignment of the light chain (> 90%). Both procedures obtained lower assignments on the heavy chain, similar to the manual-semi-automatic assignment. This region of the Fab domain showed lower spectral resolution and was more challenging.

**Table 2:** Test of *de novo* assignments using BARASA using the above the following parameters: convergence p-value: 0.99; Stepwise energy drop: -100; Min. chemical shift energy: -50

Pre-assign list	RunAbout	MIX	SHIFTX2
No. of assignments^1^	361	370	335
Matching assignments^2^	335/361 (92.8%)	328/370 (88.6%)	304/335 (90.7%)
Matching HC-Variable (117)^3^	73/81 (90.1%)	68/93 (73.1%)	67/83 (80.7%)
Matching HC-Constant (100)^3^	80/87 (92.0%)	78/87 (89.7%)	66/69 (95.7%)
Matching LC-Variable (102)^3^	90/94 (95.7%)	88/92 (95.7%)	86/94 (91.5%)
Matching LC-Constant (101)^3^	92/99 (92.9%)	94/98 (95.9%)	85/89 (95.5%)

*Note* Total number of non-proline residues (excluding the linker) = 420

1. The number of assignments corresponds to the results returned from the calculations

2. Matching assignment between BMRB 52,228 and the results returned from BARASA

3. Matching assignment between BMRB 52,228 and corresponding domains: V_H_, C_H_1, V_L_, and C_L_

**Fig. 4:**
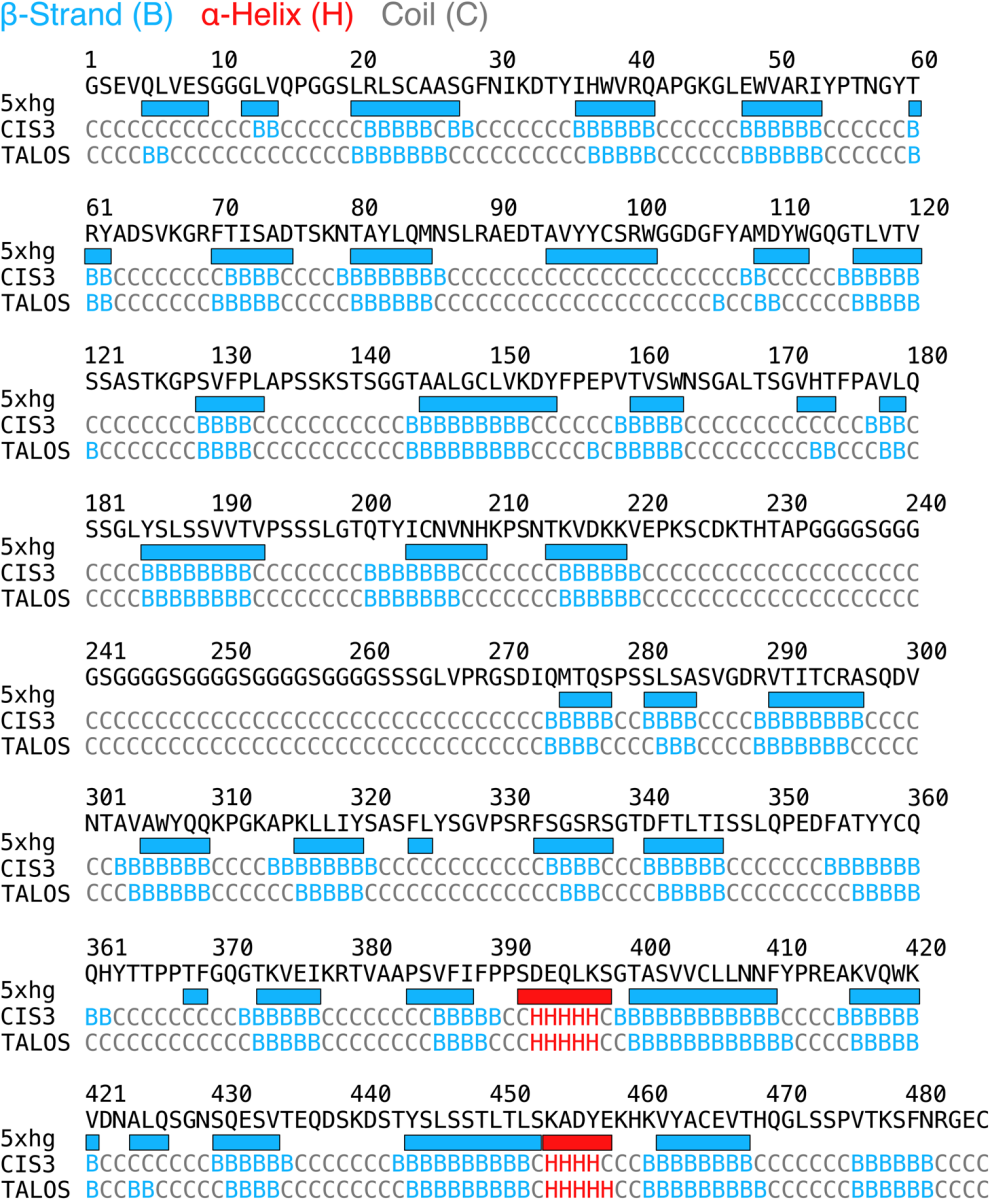
Predictions of secondary structure elements of trastuzumab-scFab from backbone chemical shift using web platforms CSI 3.0 and TALOS-N compared with the X-ray structure. Helices are labeled H (red), beta-strands are labeled B (cyan) and coils are labeled C (grey)

Prediction of secondary structure elements using CSI 3.0 and TALOS-N allowed further validation of the assignments. Predicted values of backbone torsion angles, visualized as secondary structure elements, are consistent with the X-ray structure (PDB ID 5xhg) (Fig. 4).

The near complete resonance assignment of the Fab fragment trastuzumab will contribute to a better characterization of biosimilar products for this important mAb therapeutic. In addition, it will allow the study of effects of drug product excipients on backbone amide and methyl groups dynamics.

## Data Availability

Chemical shifts and Bruker raw data ser files were deposited in the BMRB data bank with entry number 52228.

## Abbreviations

mAb: Monoclonal antibody
scFab: Single-chain fragment antigen-binding
Fab: Fragment antigen-binding
VH: Heavy chain variable domain
VL: Light chain variable domain
